# A Prospective Randomized Study on the Risk of Bacteremia in Banding versus Sclerotherapy of Esophageal Varices

**DOI:** 10.3389/fmed.2016.00016

**Published:** 2016-05-02

**Authors:** Marc J. Zuckerman, Yi Jia, Jesus A. Hernandez, Venkateswara R. Kolli, Arturo Norte, Hemal Amin, Nancy A. Casner, Alok Dwivedi, Hoi Ho

**Affiliations:** ^1^Division of Gastroenterology, Texas Tech University Health Sciences Center, El Paso, Texas, USA; ^2^Division of Biostatistics and Epidemiology, Texas Tech University Health Sciences Center, El Paso, Texas, USA; ^3^Division of Infectious Disease, Texas Tech University Health Sciences Center, El Paso, Texas, USA

**Keywords:** bacteremia, banding, esophageal varices, sclerotherapy, cirrhosis

## Abstract

**Background:**

Esophageal variceal banding may be less likely to cause bacteremia than sclerotherapy. The existing data about the frequency of bacteremia after esophageal variceal banding are conflicting, and few studies include both banding and sclerotherapy.

**Aims:**

We conducted a prospective randomized controlled trial to compare the frequency of bacteremia after esophageal variceal banding and sclerotherapy.

**Methods:**

Over a 2-year period, patients with liver disease admitted for upper gastrointestinal bleeding or for outpatient elective variceal therapy were enrolled. New patients were randomized preprocedure to either banding or sclerotherapy, and subsequent sessions utilized the initial procedure. The groups consisted of banding, sclerotherapy, and endoscopy without variceal therapy. Subjects underwent endoscopy by one out of three gastroenterologists. Blood cultures were obtained 5 min before and 30 min after endoscopy to check for bacteremia.

**Results:**

Postendoscopic blood cultures were positive following 4 out of 139 (2.9%) sessions: 1 sclerotherapy and 3 control sessions. All postendoscopic positive blood cultures were found following emergency sessions (4/92, 4.3%). One pre-endoscopic blood culture was positive in a patient with emergency banding. The rates of positive postendoscopic blood cultures among groups with emergency banding (0/22, 0%), emergency sclerotherapy (1/41, 2.3%), and emergency control (3/29, 10.3%) were not significantly different. Postendoscopic positive blood cultures were not found after elective sessions with either banding or sclerotherapy.

**Conclusions:**

Postendoscopic bacteremia was infrequent following emergency endoscopy in patients with esophageal variceal bleeding. Bacteremia was not found after esophageal variceal banding, although this was not significantly less frequent than after sclerotherapy. Postendoscopic bacteremia was not associated with elective variceal therapy.

## Introduction

Endoscopic therapy is the most reliable treatment for esophageal varices, for both active bleeding and prevention of re-bleeding (1). Endoscopic variceal ligation (EVL) has replaced endoscopic variceal sclerotherapy (EVS) as a treatment for esophageal varices (2). Bacteremia can occur after any endoscopic procedure, including diagnostic esophagogastroduodenoscopy, as the result of bacterial translocation of endogenous microbial flora into the bloodstream (3–5). There has been concern about the risk of infectious adverse events after endoscopic therapy of varices. However, the incidence of bacteremia after EVL in patients with cirrhosis or portal hypertension is reported to be low and with few adverse events (6–8). Although there have been reports of infection with both EVL and EVS procedures, the incidence of transient bacteremia after EVL (3–6%) (6–9) may be lower than that after EVS (0–53%) (9–12). Moreover, the total incidence of all types of infectious adverse events after EVL (1.8%) may be lower than for EVS (18%) (6). A retrospective study has implied that the rate of clinical bacterial peritonitis after EVL may also be lower (6). However, the existing data are conflicting due to small sample sizes and variable controls (6–11).

In current guidelines addressing the risk of bacteremia with endoscopic variceal therapy, we noted the limited data on bacteremia with EVL (5, 13–15). We previously studied the risk of bacteremia in sclerotherapy of esophageal varices and reported clinically important postendoscopic bacteremia in 10.6% of the emergency sclerotherapy sessions. After EVL was introduced, we designed this prospective randomized study to compare the rate of bacteremia after endoscopic esophageal variceal ligation with that of sclerotherapy.

## Materials and Methods

### Subjects

**Figure 1:**
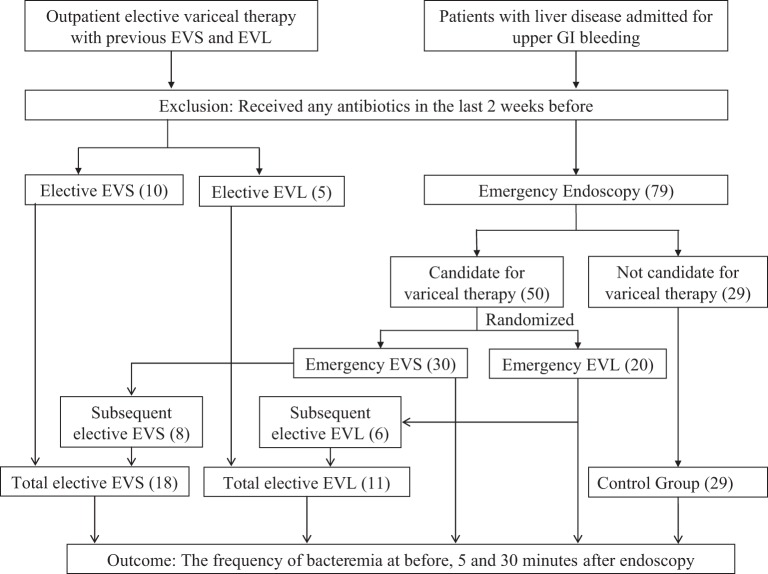
**Flowchart of the study design**. Note: EVS, endoscopic variceal sclerotherapy; EVL, endoscopic variceal ligation. The number of patients is reported in parenthesis ().

Patients with liver disease admitted for UGI bleeding or for outpatient elective variceal therapy with previous EVS and EVL at Thomason General Hospital in El Paso, TX, USA were included in the study (Figure 1). Patients were excluded if they had received any antibiotics in the last 2 weeks before inclusion in the study. Patients were enrolled and underwent endoscopy by one out of three gastroenterologists (Marc J. Zuckerman, Jesus A. Hernandez, and Venkateswara R. Kolli). New patients were randomized preprocedure to either banding or sclerotherapy, and subsequent sessions utilized the initial procedure. The study was approved by the Texas Tech University Health Sciences Center Institutional Review Board, and informed consent was obtained before patients entered the study.

A total of 139 (63 emergency variceal therapy, 47 elective variceal therapy, and 29 emergency endoscopy control) endoscopies were performed in 94 patients. The emergency groups consisted of all patients who underwent endoscopy within 24 h of admission or within 24 h of re-bleeding in hospital. The elective group consisted of all patients without UGI bleeding at the time of admission and admitted to the hospital for the follow-up endoscopic procedures. There were five groups of patients: (a) emergency endoscopy with sclerotherapy, 41 sessions in 30 patients; (b) elective endoscopy with sclerotherapy, 29 sessions in 18 patients; (c) emergency endoscopy with banding, 22 sessions in 20 patients; (d) elective endoscopy with banding, 18 sessions in 11 patients; and (e) endoscopy without banding or sclerotherapy (patients suspected of having UGI bleeding due to esophageal varices, but found to have another source, controls), 29 sessions in 29 patients.

### Endoscopy

Patients were premedicated with IV diazepam or midazolam and at times, meperidine. Endoscopy was performed with Olympus GIF endoscopes (Olympus, Lake Success, NY, USA). Sclerotherapy was performed with a 25-gage, 4-mm disposable flexible injector (Flexitip Retractable Sclerotherapy needle no 198; American Endoscopy, Inc., Mentor, OH, USA) and sodium tetradecyl sulfate 1.5% (Elkins-Sinn Inc., Cherry Hill, NJ, USA) in D50W sclerosant. Injections were administered intravariceally with an average of 2 mL of sclerosant (range, 1–3 mL) per injection. EVL was performed using an overtube and the Steigman–Goff single band ligator (Bard Interventional Products, Billerica, MA, USA). A 25-cm overtube was backloaded over the shaft of the endoscope. After the endoscope entered the esophagus, the overtube was pushed forward over the shaft of the endoscope. The endoscopic ligating device was subsequently attached to the distal end of the endoscope. Each varix was ligated with one rubber band or until the bleeding stopped. During elective sessions, individual ligation sites were gradually reduced until the varices were too small to ligate.

### Blood Cultures

Blood cultures (total 415 blood cultures obtained in 139 sessions) were drawn *via* separate venous punctures of the forearm at 5 min (BCX1) before endoscopy and 30 min (BCX3) after the endoscopy. The blood cultures were taken by the research coordinator (AN) after the patient’s forearm was cleansed with povidone-iodine and 70% isopropyl alcohol. Blood, 5 mL, was inoculated into each Trypticase Soy Broth bottle (Becton Dickinson, Towson, MD, USA) for both aerobic and anaerobic cultures. Bacterial growth was monitored radiometrically for 7 days with the Bactec 360 Microscan system (Baxter, West Sacramento, CA, USA).

### Data Analysis

Continuous data were described using mean and SD, whereas categorical data were described using frequency and proportion. Baseline characteristics were compared among groups. Continuous data were compared using the one-way analysis of variance (ANOVA), whereas categorical data were compared using Fisher’s exact test. The outcome, risk of bacteremia, was compared first looking at the risk of bacteremia between sclerotherapy with banding using Fisher’s exact test. After this, the intervention group (EVS or EVL) was compared with the control group for risk of bacteremia using Fisher’s exact test. In the secondary analysis, emergency and elective groups were also compared for risk of bacteremia using Fisher’s exact test. Finally, five groups were compared using Fisher’s exact test. All the *p*-values less than 5% were considered as significant results. Statistical analysis was carried out using STATA 12.1.

## Results

A total of 139 endoscopy sessions were performed in a total of 94 patients enrolled in the study (Table 1). The emergency variceal therapy endoscopies were performed in 63 sessions in 50 patients (41 sessions in 30 patients with EVS, and 22 sessions in 20 patients with EVL). The elective variceal therapy had 47 sessions in 29 patients (29 sessions in 18 patients with EVS and 18 sessions in 11 patients with EVL). The control groups (29 sessions in 29 patients) included emergency endoscopies performed due to suspected esophageal varices, but patients were found to have other bleeding sources (Table 2).

**Table 1:** **Characteristics of sclerotherapy, banding, and control group sessions**.

	Sclerotherapy	Banding	Control	*P*-value
Age (year)	49.1 ± 11.5	48.6 ± 12.0	50.1 ± 11.7	0.56
Sex (M/F)	65/5	29/11	26/3	0.01
Patients (*n*)	(Total 40)	(Total 25)	(Total 29)	
Emergency	30	20	29	
Elective	18	11	0	
Sessions	(Total 70)	(Total 40)	(Total 29)	
Emergency	41	22	29	–
Elective	29	18	0	–
Etiology				0.00
Alcohol	36	30	16	–
HCV	1	0	2	–
Alcohol/HCV	23	1	4	–
Alcohol/HCV/HBV	7	1	0	–
Other	3	8	9	–
Encephalopathy (A/B/C)	67/3/0	39/1/0	25/3/1	0.18
Ascites (A/B/C)	49/20/1	34/4/2	24/2/3	0.016
Bilirubin (mg/dL)	2.9 ± 4.4	2.1 ± 1.2	2.1 ± 1.7	0.34
Albumin (g/dL)	2.7 ± 0.8	2.6 ± 0.4	3.0 ± 0.9	0.14
Prothrombin time (s)	15.2 ± 2.6	15.0 ± 1.9	13.9 ± 1.9	0.054
Child-Pugh score	7.6 ± 1.9	7.6 ± 1.6	7.3 ± 2.4	0.70
Child class (A/B/C)	22/33/14	10/25/5	15/10/4	0.11
Active bleeding^a^	6.2%	5.4%	15.4%	0.27
Hematocrit (g/dL)	29.6 ± 7.2	28.9 ± 6.6	32.1 ± 7.3	0.17
Procedure time (min)	12.9 ± 4.8	16.4 ± 8.3	17.3 ± 10.7	0.018
Sclerosant vol (mL)	11.5 ± 4.8	–	–	*n/a*
Sclerosant inj (*n*)	6.1 ± 2.3	–	–	*n/a*
Bands (*n*)	–	5.2 ± 2.1	–	*n/a*

*A total of 139 endoscopies were performed in 94 patients, 29 sessions were in the emergency endoscopy control group, and 110 in the endoscopic variceal therapy groups (63 emergency variceal therapy and 47 elective variceal therapy)*.

*^a^Active bleeding was 10.5% for emergency sclerotherapy and 10.5%for emergency banding, p = 0.2 comparing the three emergency groups*.

**Table 2:** **Sources of UGI bleeding in emergency endoscopy control group (*n* = 29)**.

Source	Sessions number (%)	Associated abnormalities
PHG^a^	6 (20.7)	GU-1, esoph varices – 2
Gastric ulcer^b^	6 (20.7)	Gastritis-2, esoph varices-1, DU-1
Mallory–Weiss tear	5 (17.2)	PHG-1
Duodenal ulcer^b^	4 (13.8)	
Erosive esophagitis	4 (13.8)	Gastritis-2
Gastric erosions	3 (10.3)	Esoph varices-1
Small esophageal varices	1 (3.4)	

*^a^PHG-portal hypertensive gastropathy*.

*^b^Ulcers treated with Bicap (GU-2, DU-2)*.

*Of the 29 sessions, 17 were in patients within 24 h of admission, 6 were in patients >24 h after admission, and 6 were indeterminate*.

The sclerotherapy, banding, and control groups were comparable in age (Table 1). Most patients had a history of alcohol abuse as the main etiology of liver disease. The average Child-Pugh score was 7.6 in both the EVL and EVS groups and 7.3 in the control group. Active bleeding was 10.5% in emergency sclerotherapy, 10.0% in emergency banding, and 15.4% in the control group. Both the EVL and emergency endoscopy groups had a longer duration of procedure time than the EVS group (*p* = 0.018). The average number of injections was six in patients with sclerotherapy, and the average number of bands placed was five in the patients who had banding.

Positive postendoscopic blood cultures were found from 6 out of 415 blood cultures (14.5%) (Table 3). The bacteremia rate after endoscopic procedures was 4.3%, found in 4 out of 92 emergency sessions. For elective endoscopy sessions, no positive blood culture was found after either 18 banding or 29 sclerotherapy sessions.

**Table 3:** **Number of sessions with positive blood culture results before and after endoscopy**.

Group	BCX 1 (%)	BCX 2 (%)	BCX 3 (%)	Total after endoscopy
Emergency EVS (41)	0 (0)	1 (2)	0 (0)	1 (2)
Elective EVS (29)	0 (0)	0 (0)	0 (0)	0 (0)
Emergency EVL (22)	1 (5)	0 (0)	0 (0)	0 (0)
Elective EVL (18)	0 (0)	0 (0)	0 (0)	0 (0)
Emergency endoscopy (control) (29)	0 (0)	2 (7)	2 (7)	4 (10)

*BCX, blood culture – BCX1 (before), BCX2 (5 min), and BCX 3 (30 min)*.

There were no significant differences in the rates of postendoscopic bacteremia between the sclerotherapy (1/70) and banding (0/40) method (Table 4). The rates of bacteremia were significantly higher in the emergency control (3/29) group than the variceal therapy (1/110) groups (*p* = 0.029).

**Table 4:** **Comparisons of risk of bacteremia after endoscopy according to different groups**.

Group	Risk of bacteremia		*P*-value
	Positive	Negative	
EVS versus EVL			
EVS	1	69	1.000
EVL	0	40	
Emergency			
EVS	1	40	1.000
EVL	0	22	
Elective			
EVS	0	29	NA
EVL	0	18	
Variceal therapy versus control			0.029
Variceal therapy (EVS/EVL)	1	109	
Control	3	26	
Emergency versus elective			1.000
Emergency EVS	1	62	
Elective EVS	0	47	
Overall comparison			0.162
Emergency EVS	1	40	
Elective EVS	0	29	
Emergency EVL	0	22	
Elective EVL	0	18	
Emergency endoscopy (control)	3	26	

The rates of bacteremia were not significantly different between emergency variceal therapy groups (1/63) and elective variceal therapy groups (0/47). There were also no significant differences in the rates of bacteremia comparing the five different study groups, emergency EVS (1/41), elective EVS (0/29), emergency EVL (0/22), elective EVL (0/18), and emergency control (3/29).

The organisms isolated from each positive blood culture obtained either before or after endoscopy are listed in Table 5. One positive blood culture (*Strep. intermedius*) was obtained pre-endoscopy in a patient randomized to emergency banding. Five blood cultures from 4 patients in 139 sessions (2.9%) were positive. All the patients who had bacteremia after endoscopy were Child Class B. Active bleeding was present in one of the four patients.

**Table 5:** **Bacteria isolated from blood culture before and after endoscopy**.

Patient	BCX 1	BCX 2	BCX 3
Emergency EVS			
1 (144)	–	*S. aureus*	–
Emergency EVL			
1 (010)	*S. intermedius*	–	–
Emergency endoscopy			
1 (056)	–	*E. coli*	
2 (081)	–	–	*S. epidermidis*
3 (095)	–	*S. epidermidis*	*S. epidermidis*

*Of 415 blood cultures obtained (in 139 sessions), 6 were positive (in 5 sessions)*.

*BCX, blood cultures – BCX1 (before), BCX2 (5 min), and BCX 3 (30 min)*.

*Positive postendoscopic blood cultures were: one in an emergency sclerotherapy patient (*Staph. aureus*) and three in control patients (*E. coli* in one and *Staph. epidermidis* in two). One blood culture was positive pre-endoscopy in a patient randomized to emergency banding (*Strep. intermedius*)*.

Of these positive cultures, one was in an emergency sclerotherapy session (*Staph. aureus*) and three were in emergency endoscopy control sessions (*E. coli* in one, *Staph. epidermidis* in two). Although we do not consider these to be patients with definite identifiable sources of infection, we summarize the clinical context for each patient. One patient had positive cultures of *Staph. epidermidis* at both 5 and 30 min. This patient had redness in the wrist at the intravenous access site without infectious complications after the procedure. The other patient with one positive culture of *Staph. epidermidis* developed pneumonia with sputum culture positive for *Strep. pneumonia*. These positive cultures of *Staph. epidermidis* may be contaminants. The patient with positive culture of *E. coli* had abdominal pain and leukocytosis on admission before endoscopy and developed culture negative spontaneous bacterial peritonitis (SBP) after endoscopy. Although it is not clear whether the detected *E. coli* is clinically significant or the cause of SBP, the endoscopic procedure, is unlikely to be the source and the bacteremia may be secondary to pre-existing SBP. One patient in the emergency EVS group had a positive culture of *Staph. aureus*, which is likely to be a nosocomial infection with source undetected.

## Discussion

In our prospective randomized controlled trial, postendoscopic bacteremia was infrequent following emergency endoscopy in patients with esophageal variceal bleeding. Bacteremia was not found after esophageal variceal banding, although this was not significantly less frequent than after sclerotherapy. Postendoscopic bacteremia was not associated with elective variceal therapy.

Patients with chronic liver disease and endoscopic procedures for esophageal varices are predisposed to bacteremia and infections (1, 16, 17). Several mechanisms of bacterial contamination have been proposed for endoscopy-related bacteremia (18, 19). Bacteremia has been detected as the transient contamination of oral or digestive pathogens (20), potential transluminal seeding from the EVS needle, a contamination of the side channel of the endoscope, or a contamination of sclerosants (19). The acute variceal hemorrhage itself can also provide possible mechanisms of bacterial contamination (18). The exposure to sclerosants is recognized as a foreign material and provides an ideal route for bacterial invasion, whereas infected sclerosants could form an anatomic path between the gastrointestinal lumen and the vascular spaces (19).

Many cases of bacteremia post EVS and EVL may be due to bacterial contamination from a skin source, such as *Strep. pyogenes*, *Diphtheroid* species, and coagulase-negative *Staphylococcus* (9–11, 21). Coagulase-negative *Staphylococcus* is one of the most frequent causes of nosocomial bloodstream infection and may be found in patients with no clinically significant presentations (22). We reported three *Staph. epidermidis* blood cultures in two patients. Prior to endoscopy, one patient randomized to the emergency EVL group had *Strep. intermedius*, which is a commensal organism that can be found in the oral cavity and gastrointestinal tract and is more prevalent in the saliva of patients with alcohol abuse (23). Although we report a higher bacteremia rate in the emergency endoscopy without variceal therapy (control) group (10.3%) compared with the emergency EVS group (2.3%), this may not be clinically significant bacteremia.

We recently published a meta-analysis on bacteremia after both EVL and EVS and found only limited data on bacteremia after EVL (24). The data in this study had not yet been reported and so was not included in the meta-analysis. There have been reports of transient bacteremia after both EVL and EVS (6, 8, 16, 21, 25). The incidence of infectious adverse events after EVL may be lower than that for EVS, although the existing data are conflicting. Early studies reported higher transient bacteremia in the EVS group than the EVL group that was statistically significant, 17.2% in EVS compared with 3.3% in EVL (*p* < 0.03) (6), or not statistically significant, 40% in EVS compared with 25% in EVL (16). However, most recent studies have found higher rates of positive blood cultures in EVL compared with EVS group [5.7% in EVL compared with 4.6% in EVS (25), 4.6% in EVL compared with 0.0% in EVS (21)]. In the present study, postendoscopic bacteremia was detected only in emergent EVS and emergency endoscopy without variceal therapy groups (10.3%), but not in EVL group.

The risks of transient bacteremia are different in emergency and elective procedures (11, 16). In general, patients with acute presentations are usually sicker. Patients with active or recent bleeding may have variceal walls more susceptible to bacterial invasion. In one study, none of the patients developed bacteremia after the elective cyanoacrylate injection for EVS, whereas bacteremia was significantly higher in patients who presented for emergency EVS to control variceal bleeding (15% at 5 min and 10% at 3 h) (26). Our study detected postendoscopic bacteremia only in emergency sessions, but not in any elective sessions. The difference may be real but could be due to the insufficient numbers of subjects in the studies.

Although some studies conducted the blood culture at different time frames, such as longer after endoscopic therapy, most previous studies detected positive blood cultures within 1 h after endoscopy. Therefore, our study design for blood cultures at 5 min (BCX2) and 30 min (BCX3) after the endoscopy was rational to detect the bacteremia after endoscopy.

Compared with EVL, EVS has been associated with a higher risk of various adverse events, including pleuropulmonary, bleeding, and infective events (27–29). In the present study, we did not find significant complications after endoscopic procedures, except pneumonia with a positive sputum culture in one patient. One patient had abdominal pain and leukocytosis before endoscopy and developed culture negative SBP in the hospital after endoscopy, which is may due to pre-existing SBP.

Limitations of our study include the relatively small numbers of patients in each group and limited power for the demonstration of statistically significant differences. This study was initiated soon after the introduction of the single band ligator and, therefore, did not study the risk of bacteremia after EVL using current multiband ligators (30, 31). However, most studies of bacteremia after banding have been done with single band ligators, so that the data on bacteremia after multiband esophageal ligation are limited, and none of the studies utilize the current equipment popular in the United States.

In conclusion, our findings are consistent with a low risk of bacteremia after EVL. We found no bacteremia after either elective or emergency variceal ligation. Bacteremia occurred in emergency sclerotherapy, but was infrequent, and we found no bacteremia after elective sclerotherapy. These results add more information for use in guidelines on the risk of bacteremia after variceal ligation (5, 13, 14).

## Author Contributions

MZ: conception and design, analysis and interpretation of the data, and drafting of the article; YJ: analysis and interpretation of the data, and drafting of the article; JH: conduct study; AN: conduct study, analysis, and interpretation of the data; HA: analysis and interpretation of the data; NC: organize the study; AD: analysis and interpretation of the data; HH: conception and design, critical revision of the article for important intellectual content, analysis, and interpretation of the data. All authors read and approved the final manuscript.

## Conflict of Interest Statement

The authors declare that the research was conducted in the absence of any commercial or financial relationships that could be construed as a potential conflict of interest.

## Abbreviations

EVL, esophageal band ligation; EVS, esophageal variceal sclerotherapy; UGI, upper gastrointestinal.

## References

[1] Riggio O Angeloni S Nicolini G Merli M Merkel C Endoscopic screening for esophageal varices in cirrhotic patients. Hepatology (2002) 35(2):501–2. 10.1053/jhep.2002.31308 11826432

[2] Laine L Cook D. Endoscopic ligation compared with sclerotherapy for treatment of esophageal variceal bleeding. A meta-analysis. Ann Intern Med (1995) 123(4):280–7. 10.7326/0003-4819-123-4-199508150-00007 7611595

[3] Lin OS Wu SS Chen YY Soon MS. Bacterial peritonitis after elective endoscopic variceal ligation: a prospective study. Am J Gastroenterol (2000) 95(1):214–7. 10.1111/j.1572-0241.2000.01687.x 10638586

[4] Nelson DB Infection control during gastrointestinal endoscopy. Can J Gastroenterol (2007) 21(1):13–5. 10.1155/2007/169846 17290545 PMC2656624

[5] Banerjee S Shen B Baron TH Nelson DB Anderson MA Cash BD Antibiotic prophylaxis for GI endoscopy. Gastrointest Endosc (2008) 67(6):791–8. 10.1016/j.gie.2008.02.068 18374919

[6] Lo GH Lai KH Shen MT Chang CF. A comparison of the incidence of transient bacteremia and infectious sequelae after sclerotherapy and rubber band ligation of bleeding esophageal varices. Gastrointest Endosc (1994) 40(6):675–9. 7859963

[7] Tseng CC Green RM Burke SK Connors PJ Carr-Locke DL. Bacteremia after endoscopic band ligation of esophageal varices. Gastrointest Endosc (1992) 38(3):336–7. 10.1016/S0016-5107(92)70427-1 1607085

[8] Berner JS Gaing AA Sharma R Almenoff PL Muhlfelder T Korsten MA. Sequelae after esophageal variceal ligation and sclerotherapy: a prospective randomized study. Am J Gastroenterol (1994) 89(6):852–8. 8198093

[9] Sauerbruch T Holl J Ruckdeschel G Forstl J Weinzierl M. Bacteriaemia associated with endoscopic sclerotherapy of oesophageal varices. Endoscopy (1985) 17(5):170–2. 10.1055/s-2007-1018492 4054061

[10] Camara DS Gruber M Barde CJ Montes M Caruana JA Jr Chung RS. Transient bacteremia following endoscopic injection sclerotherapy of esophageal varices. Arch Intern Med (1983) 143(7):1350–2. 10.1001/archinte.1983.00350070066013 6870408

[11] Ho H Zuckerman MJ Wassem C. A prospective controlled study of the risk of bacteremia in emergency sclerotherapy of esophageal varices. Gastroenterology (1991) 101(6):1642–8. 1955129 10.1016/0016-5085(91)90403-8

[12] Brayko CM Kozarek RA Sanowski RA Testa AW. Bacteremia during esophageal variceal sclerotherapy: its cause and prevention. Gastrointest Endosc (1985) 31(1):10–2. 10.1016/S0016-5107(85)71955-4 3872240

[13] Qureshi W Adler DG Davila R Egan J Hirota W Leighton J ASGE Guideline: the role of endoscopy in the management of variceal hemorrhage, updated July 2005. Gastrointest Endosc (2005) 62(5):651–5. 10.1016/j.gie.2005.07.031 16246673

[14] Garcia-Tsao G Sanyal AJ Grace ND Carey WD Prevention and management of gastroesophageal varices and variceal hemorrhage in cirrhosis. Am J Gastroenterol (2007) 102(9):2086–102. 10.1111/j.1572-0241.2007.01481.x 17727436

[15] Hwang JH Shergill AK Acosta RD Chandrasekhara V Chathadi KV Decker GA The role of endoscopy in the management of variceal hemorrhage. Gastrointest Endosc (2014) 80(2):221–7. 10.1016/j.gie.2013.07.023 25034836

[16] Kulkarni SG Parikh SS Dhawan PS Chachad H Jambavalikar MB Koppikar GV High frequency of bacteremia with endoscopic treatment of esophageal varices in advanced cirrhosis. Indian J Gastroenterol (1999) 18(4):143–5. 10651544

[17] Maulaz EB de Mattos AA Pereira-Lima J Dietz J. Bacteremia in cirrhotic patients submitted to endoscopic band ligation of esophageal varices. Arq Gastroenterol (2003) 40(3):166–72. 10.1590/S0004-28032003000300006 15029392

[18] Rerknimitr R Chanyaswad J Kongkam P Kullavanijaya P. Risk of bacteremia in bleeding and nonbleeding gastric varices after endoscopic injection of cyanoacrylate. Endoscopy (2008) 40(8):644–9. 10.1055/s-2008-1077294 18561097

[19] Wahl P Lammer F Conen D Schlumpf R Bock A Septic complications after injection of N-butyl-2-cyanoacrylate: report of two cases and review. Gastrointest Endosc (2004) 59(7):911–6. 10.1016/S0016-5107(04)00341-4 15173814

[20] Chen WC Hou MC Lin HC Yu KW Lee FY Chang FY Bacteremia after endoscopic injection of N-butyl-2-cyanoacrylate for gastric variceal bleeding. Gastrointest Endosc (2001) 54(2):214–8. 10.1067/mge.2001.116566 11474393

[21] Bonilha DQ Correia LM Monaghan M Lenz L Santos M Libera ED. Prospective study of bacteremia rate after elective band ligation and sclerotherapy with cyanoacrylate for esophageal varices in patients with advanced liver disease. Arq Gastroenterol (2011) 48(4):248–51. 10.1590/S0004-28032011000400006 22147129

[22] Rogers KL Fey PD Rupp ME. Coagulase-negative staphylococcal infections. Infect Dis Clin North Am (2009) 23(1):73–98. 10.1016/j.idc.2008.10.001 19135917

[23] Morita E Narikiyo M Yokoyama A Yano A Kamoi K Yoshikawa E Predominant presence of Streptococcus anginosus in the saliva of alcoholics. Oral Microbiol Immunol (2005) 20(6):362–5. 10.1111/j.1399-302X.2005.00242.x 16238596

[24] Jia Y Dwivedi A Elhanafi S Ortiz A Othman M Zuckerman M Low risk of bacteremia after endoscopic variceal therapy for esophageal varices: a systematic review and meta-analysis. Endosc Int Open (2015) 03(05):E409–17. 10.1055/s-0034-1392552 26528494 PMC4612236

[25] da Silveira Rohr MR Siqueira ES Brant CQ Morais M Libera ED Jr Castro RR Prospective study of bacteremia rate after elastic band ligation and sclerotherapy of esophageal varices in patients with hepatosplenic schistosomiasis. Gastrointest Endosc (1997) 46(4):321–3. 10.1016/S0016-5107(97)70118-4 9351034

[26] Maluf-Filho F Sakai P Ishioka S Matuguma SE. Endoscopic sclerosis versus cyanoacrylate endoscopic injection for the first episode of variceal bleeding: a prospective, controlled, and randomized study in Child-Pugh class C patients. Endoscopy (2001) 33(5):421–7. 10.1055/s-2001-14257 11396760

[27] Truesdale RA Jr Wong RK. Complications of esophageal variceal sclerotherapy. Gastroenterol Clin North Am (1991) 20(4):859–70. 1787018

[28] Bac DJ de Marie S Siersema PD Snobl J van Buuren HR. Post-sclerotherapy bacterial peritonitis: a complication of sclerotherapy or of variceal bleeding? Am J Gastroenterol (1994) 89(6):859–62. 8198094

[29] Edling JE Bacon BR. Pleuropulmonary complications of endoscopic variceal sclerotherapy. Chest (1991) 99(5):1252–7. 10.1378/chest.99.5.1252 2019188

[30] El-Saify WM Mourad FA. Use of the six-shooter ligation device in the management of bleeding esophageal varices: a developing-country experience. Dig Dis Sci (2005) 50(2):394–8. 10.1007/s10620-005-1617-x 15745107

[31] Woods KL Qureshi WA. Long-term endoscopic management of variceal bleeding. Gastrointest Endosc Clin N Am (1999) 9(2):253–70. 10333441

